# Implications for the Ergogenic Benefits of Self-Selected Music in Neurological Conditions: A Theoretical Review

**DOI:** 10.3390/neurolint17070106

**Published:** 2025-07-11

**Authors:** Christopher G. Ballmann, Rebecca R. Rogers, Sophia L. Porrill, Nicholas B. Washmuth

**Affiliations:** 1 Department of Human Studies, University of Alabama at Birmingham, Birmingham, AL 35294, USA; 2 Department of Physical Therapy, University of Alabama at Birmingham, Birmingham, AL 35209, USA; 3 Department of Family and Community Medicine, University of Alabama at Birmingham, Birmingham, AL 35294, USA; rrrogers@uab.edu; 4 Department of Psychology, The University Alabama in Huntsville, Huntsville, AL 35899, USA; nw0077@uah.edu

**Keywords:** music preference, exercise, rehabilitation, disability

## Abstract

The ergogenic effects of music have been well described across various modes of exercise with widespread use across competitive athletes and recreational exercisers alike. Underlying the acute beneficial effects of music during exercise are profound physiological and psychological changes which involve an array of different organ systems, including but not limited to cardiovascular, endocrine, skeletal muscle, and nervous systems. While the use of music to enhance physical performance and improve associated mechanisms has been largely optimized in healthy individuals, the investigations of the translation to individuals with neurological conditions are still ongoing. Recently, it has been established that the personalization of music interventions greatly influences performance-enhancing benefits and aids in physical performance optimization in healthy individuals. Self-selected music (SSM) has been documented to impart ergogenic advantages over pre-determined or non-preferred music, including improved cardiorespiratory endurance, power development, and velocity of movement which are characterized by adaptative physiological and psychological changes. Evidence of the benefits of SSM has progressed to the degree to which the overlap of possible benefits between healthy and clinical populations is becoming more apparent. This aim of this theoretical review is to discuss how personalized music influences psychophysiological determinants of exercise ability in healthy individuals and consider how these findings may be applicable to neurological conditions to enhance exercise capacity. The current knowledge on the role of SSM in augmenting physiological and psychological responses to exercise in healthy individuals is presented along with how these mechanisms might be leveraged to overcome exercise limitations in neurological conditions. Overall, SSM appears to have theoretical support to be a promising therapeutic approach to improving exercise ability in neurological conditions through similar ergogenic mechanisms documented in healthy individuals, but further investigation is warranted.

## 1. Introduction

Music has been well established as an effective ergogenic aid and is widely used by the general population, including competitive athletes and recreational exercisers. Enhanced performance and psychological responses have been noted in virtually all exercise modalities including aerobic endurance, resistance, sprints, and explosive movements [1]. Underpinning the benefits of music are adaptative physiological and psychological changes that can act independently or synergistically, contributing to the acute enhancement of physical ability [2]. Music exerts beneficial effects on various biological systems during exercise, including neural, skeletal muscle, cardiovascular, and endocrine systems [1]. Furthermore, various psychological processes are altered with music during exercise, including emotion, affect, perception, and stress [3]. These effects culminate into ergogenic benefits likely dependent on the coordination and concomitant activation of physiological and psychological processes to produce an optimal performance. Because of this, the implementation of music in exercise rehabilitation and conditions resulting in diminished exercise ability has become increasingly apparent as a complementary therapy. Ergogenic benefits of music have been shown in some neurodegenerativeand neuromuscular diseases with similar underlying physiological and psychological improvements. Despite the widespread characterization of the ergogenic benefits of music in healthy populations, the understanding of the underpinnings of music interventions in neurologic conditions remains incomplete and has yet to be clearly theorized for clinical settings.

In recent years, self-selection and music preferences have emerged as an important consideration in how music influences exercise performance and ability. Indeed, the degree to which an individual likes or dislikes various factors of music (e.g., genre, loudness, tempo) grossly mediates the physical performance and responses during exercise. Physiologically, self-selected music (SSM) has been shown to improve the neural drive, autonomic control, muscle activation, and fatigue [1,4,5,6]. Psychologically, SSM improves motivation, dissociation, arousal, and mood [7,8,9]. Thus, the efficacy of and responses to music interventions are highly dependent on the preference or self-selection by the individual listener. Many of these mechanisms mediate deficits in exercise ability in neurological conditions, and SSM may aid in overcoming barriers to the incorporation of exercise into therapeutic practice. Thus, identifying the evidence of theoretical overlaps between physiological and psychological benefits of SSM in healthy populations versus populations with neurologic conditions may improve the translational understanding of music to the clinic to progress closer to optimized music medicine interventions. The purpose of this theoretical review is to discuss how the ergogenic effects of SSM mediate physiological and psychological benefits in healthy individuals and provide available evidence of how these findings may translate to neurological conditions to improve exercise ability. The current knowledge on how SSM influences physiological and psychological responses to exercise will be discussed, followed by evidence of how these mechanisms may translate to neurological conditions to potentially improve pathological limitations to exercise. Future directions and research needs will be presented to provoke future inquiry and insight.

## 2. Ergogenic Mechanisms of Self-Selected Music

**Figure 1:**
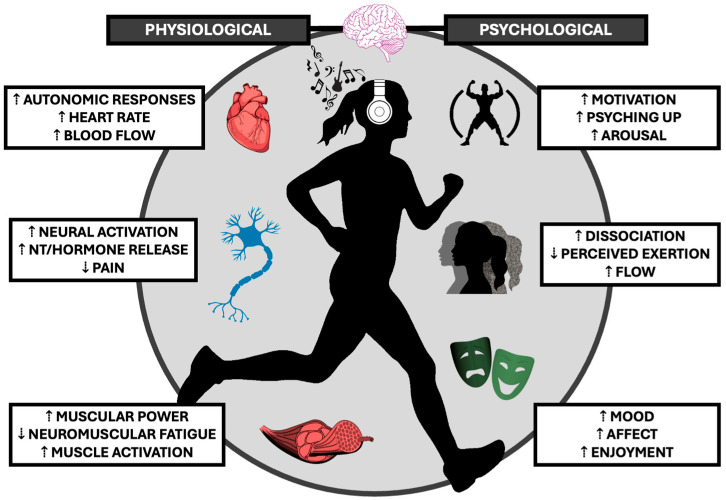
Personalizing music choices during exercise results in pronounced physiological and psychological benefits to exercise ability. Benefits span across organ systems, including cardiovascular, skeletal muscle, and nervous systems. Alterations in psychophysiological responses may be due to independent or synergistic actions, which collectively result in an enhanced exercise ability.

The purpose of the following section is to provide a foundational overview of the current knowledge of SSM and exercise performance in the context of healthy individuals. Physical performance, physiological, and psychological mechanisms mediating performance-enhancing benefits (Figure 1) will be reviewed. For a more comprehensive review of music preference and exercise performance, refer to [1]. This section is intended to be brief and provide knowledge necessary to understand the translation of ergogenic mechanisms to theoretical benefits to neurological conditions.

### 2.1. Physiological

SSM has been implicated to alter numerous physiological processes during exercise which are important to performance, including the following: (1) neural drive/activation, (2) autonomic control, and (3) fatigue indices. While the enhancements of other mechanisms exist that are beyond the scope of this review, these key mechanisms have been suggested to have the highest potential for modulation via SSM and will be the focus of the subsequent section.

It has been well described that SSM activates areas of the brain important for motor control and urges to move [10,11]. SSM has been suggested to result in stronger auditory-motor coupling, especially in supplementary motor areas compared to unknown music [10]. The neural synchronization from music to movement has been well described, which may improve exercise efficiency [12,13]. Furthermore, SSM may result in improved neural encoding and sensory processing, leading to an improved learning and control of movement [14]. Groove music, or music that induces the pleasurable urge to move, stimulates motor cortex areas, both primary and reward-associated, which improves motor task learning [15,16]. This is further supported by exercise performance research showing that SSM increases muscular power and movement velocity, suggesting the greater coordination or recruitment of motor units through motor activation [4,7,8,17].

SSM has been described to alter autonomic activity both before, during, and after exercise [18,19]. Since SSM has been suggested to induce the release of catecholamines, it is likely that SSM-induced sympathetic activation is a primary ergogenic mechanism. Indeed, listening to SSM during exercise has been shown to result in greater sympathetic dominancy and physiological excitation [20]. Listening to arousing music during a warm-up has been shown to elevate catecholamine levels, potentially enhancing muscle activation and metabolic responses during the subsequent exercise [18]. The SSM-induced catecholamine release may also result in cardiac stimulation, improving the cardiac output and blood flow delivery, although this is not fully confirmed at this time. On the contrary, non-SSM has been suggested to result in maladaptive stress responses, including a heightened adrenocorticotropic hormone (ACTH) release and anxiogenesis [21]. SSM has been shown to improve responses to physical stress, such as lower blood pressure responses to isometric exercise [21]. However, responses to SSM seem to depend on whether the music is considered stimulative or relaxing. Relaxing music has been shown to decrease norepinephrine levels, while fast-tempo music my increase epinephrine levels with exercise, suggesting a possible “double-edged sword” effect where SSM may induce stimulation or recovery [18]. This has been confirmed repeatedly by other studies showing disparate changes in the heart rate variability (HRV), a measure of autonomic branch dominancy, between varying types of stimulative or relaxing music [18,19].

More recently, music has been implicated in modifying physiological markers of fatigue from physical exertion. Stimulating music has been shown to improve neuromuscular fatigue thresholds [22]. This may be in part due the improved neuromuscular coupling and synchronization in response to the rhythm from SSM [10]. A myriad of studies have supported this, showing lower power output loss and fatigue indexes during repeated high-intensity exercise while listening to SSM [1,5,23]. The attenuation of fatigue from SSM has been suggested to be directly and indirectly related to exercise metabolism. SSM may increase the cardiac output and oxygen consumption during steady-state exercise compared to no music [24]. This could in turn lead to an improved blood flow and oxygen delivery to active skeletal muscle during physical exertion, which may improve fatigue indices. Ghaderi et al. found that trained handball players exhibited lower blood lactate levels after high-intensity exercise when listening to motivational music compared to no music [25]. Similarly, listening to music during recovery has been linked to increased lactate clearance in active males [26]. If indeed music does promote hyperemia to active muscles, this could enhance acute recovery and contribute to its ergogenic benefits, particularly in repeated exercise bouts.

### 2.2. Psychological

Numerous psychological mechanisms have been proposed to explain the ergogenic effects of SSM, with the most widely studied including (1) motivation and arousal, (2) dissociation and perceived effort, and (3) affect and enjoyment. While other mechanisms may contribute, these key factors have been suggested to hold the greatest potential for clinical application and will be the focus of the subsequent section.

Music has been widely recognized as a potent psychological tool that can enhance motivation, alter perceptions, and optimize emotional states during exercise. One of the primary mechanisms through which SSM exerts its ergogenic effects is motivation and arousal modulation. Listening to SSM has been shown to increase confidence and self-efficacy, contributing to a “psyching-up” effect that enhances the readiness for physical exertion [6,27]. Alternatively, SSM has also been reported to alleviate anxiety, promote relaxation, and enhance competitive performance [27]. Furthermore, preferred music fosters an overall more positive psychological state, which is essential for sustaining effort and optimizing performance outcomes.

Music-induced arousal regulation is another key mechanism influencing exercise performance [28]. Musical characteristics such as the tempo, rhythm, and volume have been shown to modulate arousal levels, thereby influencing physical output. For instance, listening to music before exercise has been demonstrated to increase subjective feelings of power, with effects dependent on the intensity and structure of the music [29]. Similarly, Chtourou et al. found that music-enhanced warm-ups resulted in a heightened vigor and improved anaerobic performance in sprinters [30]. The research further supports that music enhances vigor in resistance-trained individuals, improving the movement velocity and force production in ballistic exercises [31]. These findings suggest that music may prime the nervous system, optimizing the psychological readiness for performance.

Dissociation and perceived effort modulation is another well-documented mechanism underlying the benefits of SSM. Listening to SSM has been shown to lower ratings of perceived exertion (RPE) across various exercise modalities. For example, Nakamura et al. reported a significantly lower RPE in endurance cycling when SSM was present [32]. Similar reductions in RPE have been observed during high-intensity repeated sprints and resistance exercises [7,33]. The primary mechanism for this effect is believed to be attentional reallocation, whereby music shifts the focus away from physical discomfort and exertion [34]. Neurophysiological evidence suggests that music-driven attentional shifts alter cortical activity, further contributing to a reduction in RPE [35].

Listening to music plays a crucial role in affective responses and enjoyment with exercise [36]. Positive affective states have been associated with greater endurance and an improved resistance-based performance [37,38]. Building on this, Hutchison et al. found that adding SSM further enhanced positive emotions during exercise, allowing individuals to maintain a higher intensity while preserving their affective state [39]. Similarly, Elliot et al. reported that motivational music led to an increased workload completion and greater improvements in the positive affect during cycling [40]. These findings highlight the role of SSM in amplifying the affective benefits of exercise, suggesting that SSM can enhance the positive affect during exercise, even under conditions of an increased intensity and workload.

Despite the numerous psychological benefits of SSM, individual responses vary due to factors such as the music preference, training status, and exercise context. Research suggests that SSM has a greater impact on affect and RPE than non-preferred or generic music, reinforcing the importance of personalization in music selection [7,41]. The ergogenic and affective responses to music also appear to differ between trained and untrained individuals, with untrained populations exhibiting greater improvements in positive affect during exercise [42]. This may be attributed to physiological adaptations in trained individuals, who develop a higher tolerance to high-intensity exertion. Furthermore, motivational music has been shown to elicit positive affective responses, increase enjoyment, reduce RPE, and lower the metabolic demand in exercise-resistant populations [43]. These findings suggest that SSM may hold particular relevance in neurological conditions, where individuals often exhibit a lower exercise tolerance and may derive greater psychological and physiological benefits from music interventions.

## 3. Implications for Self-Selected Music as Medicine in Neurological Conditions

**Figure 2:**
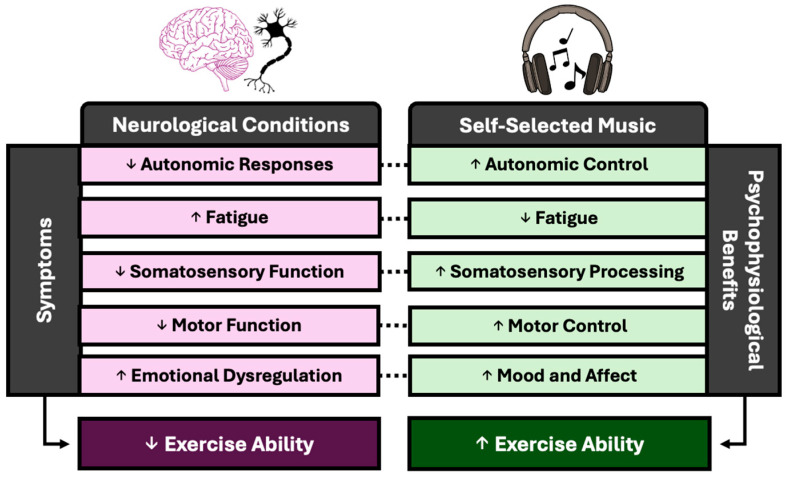
Neurological conditions result in a variety of symptoms (purple) which span various psychophysiological processes and result in a diminished exercise ability. Self-selected music (SSM) has been shown to beneficially modify a myriad of psychophysiological processes in healthy individuals (green), which may translate to the combatting of symptomology, resulting in the translation of an improved exercise ability in neurological conditions (dotted lines).

While SSM and exercise ability have been well studied in healthy populations, the translation to individuals with neurological conditions has yet to be fully realized. Benefits from SSM span a wide range of physiological and psychological mechanisms, which uniquely overlap with those of various neurological conditions (Figure 2). Thus, the strategic use of SSM as a complementary therapy in clinical populations with a relevant symptomology represents a novel and practical means to enhance rehabilitation and exercise training. The purpose of this section is to present evidence of how the ergogenic benefits of preferred music may theoretically combat common symptoms which may be limiting for exercise and rehabilitation and what future direction is needed for implementing preferred music as medicine.

### 3.1. Dysautonomia

The dysfunction of the ANS, or dysautonomia, is implicated in a wide variety of neurological conditions, including postural orthostatic tachycardic syndrome (POTS), multiple systems atrophy, Parkinson’s disease (PD), and autonomic neuropathy [44,45,46,47,48]. During exercise, dysautonomia can result in hypotension, cardiac insufficiency, syncope, and fatigue, which can serve as barriers to the initiation of exercise or lead to the pre-mature termination of exercise [44,46,48]. Thus, interventions which acutely improve exercise ability may lead to better health outcomes and progression during rehabilitation.

SSM has been shown to alter both central and peripheral psychophysiological processes related to autonomic function. Indeed, SSM has been shown to increase the activation of limbic and paralimbic regions of the brain, which are connected to emotional processing and arousal [49,50]. In particular, connections between limbic regions (e.g., ventral tegmental area, nucleus accumbens, striatum), which aid in the processing of pleasure and reward, and paralimbic structures (e.g., amygdala, hypothalamus), involved in emotional processing and stress responses, have been implicated in mediating autonomic control [50,51]. SSM has been shown to increase the functional connectivity of these areas which potently alter autonomic actions. Greater dopamine releases in limbic areas with SSM have been reported and were concomitant with psychophysiological changes, including cardiovascular and autonomic function [51]. Peripherally, SSM may alter blood pressure, arterial blood flow, and catecholamine/stress hormone release, which are tightly coupled to the autonomic state [1,52].

In healthy individuals, SSM has been shown to increase the heart rate and sympathetic dominancy during exercise [20]. This could be of clinical importance in neurological conditions where chronotropic incompetence and cardiac insufficiency are apparent. Since lower heart rates and a lack of sympathetic stimulation may lead to a supply–demand mismatch between the oxygen/nutrient need and cardiac performance, SSM may provide adaptative sympathetic stimulation during exercise, thereby increasing the cardiac output and shunting the arterial blood flow to working skeletal muscles and the brain [53,54,55]. Conversely, SSM which is relaxing in nature may increase parasympathetic activity and improve recovery. Relaxing music has been shown to lower humoral levels of norepinephrine and lactate, indicating lower metabolic stress [56]. Increased parasympathetic activity to the heart has also been denoted while listening to SSM perceived as relaxing, which is evidenced by higher heart rate variability scores and perceived relaxation [19,57]. Thus, SSM may play a dual role in autonomic control during and after exercise, whereby the perception of the stimulation or relaxation of SSM may induce sympathetic or parasympathetic dominancy. Future studies should investigate how these effects can be leveraged to improve the exercise ability and recovery in neurological conditions with autonomic dysfunction.

### 3.2. Somatosensory Dysfunction

The disruption of sensory processing, especially somatic stimuli, alters the perception of pain, touch/vibration, and proprioception. Somatosensory dysfunction is implicated in several neurological conditions with limited exercise ability, including Parkinson’s disease, Multiple Sclerosis, peripheral neuropathy, and neurological ischemia/ischemic injury [58,59,60]. Manifestations of dysfunctional somatosensory processing during exercise are often observed as hyperalgesia, hyperesthesia, poor motor coordination, and fatigue [61,62]. Since somatosensory function is critical in recognizing body movement, discomfort, and the physiological feedback of exercise intensity, deficits from neurological conditions have implications for impaired exercise ability.

Somatosensory function has been repeatedly shown to be modulated by SSM through sensory and motor pathways. Music induces the localization of activity in the auditory, motor, and somatosensory cortex areas of the brain, suggesting the concomitant regulation of responses to somatosensory and auditory stimuli [63]. The activation of these regions is linked to the emotional state and processing of the neo- and motor cortices of the brain [64]. SSM with rhythmic characteristics has been shown to improve the processing of body movement recognition and provide organization for motor planning [65,66]. The use of rhythmic music has shown a preliminary efficacy in improving proprioception and motor control, thus decreasing fall and injury risks in neurological diseases such as Parkinson’s disease, which may translate to others [67,68]. Nociceptive and pain processing have also been shown to be altered by SSM, which is evidenced by a lower anterior cingulate cortex activation with simultaneous improvements in the pain threshold and tolerance [69,70]. Given that pain is a primary limiter to exercise in many neurological conditions, improving pain thresholds may especially provide value for allowing individuals to accumulate adequate amounts of exercise volume during training to receive optimal benefits. SSM may lead to higher levels of dissociation during exercise, which can lower discomfort and perceptions of exertion [1,7,23]. SSM also decreases perceptions of fatigue and may alter motor unit fatigue thresholds, allowing for a greater work capacity by the skeletal muscle [22,71]. The lowering of fatigue and perceived exertion may also improve the accumulation of exercise volume and improve the overall tolerance to exercise interventions. However, it is important to note that gross disturbances to perceptions of fatigue and exertion may also pose safety concerns—in that overtraining or overexertion may exacerbate aspects of conditions which should be considered in future investigations.

### 3.3. Emotional Dysregulation and Affective Disturbance

Affective disturbances, such as anxiety, depression, and stress, can significantly impact physical ability and exercise tolerance. Emotional dysregulation has been noted in a variety of neurological conditions, including cardiovascular disease, psychiatric disorders, Parkinson’s disease, and neurological pain, which can be exacerbated over time by aging. Neurological conditions often result in changes to the non-motor brain pathway activity, including limbic areas that mediate psychophysiological function [72,73,74]. Limbic structure dysfunction has been implicated in multiple neurological conditions, including the ventral tegmental area and nucleus accumbens, which regulate reward-seeking and motivated behavior [75,76,77]. Paralimbic pathway dysfunction through the amygdala and hypothalamus, which largely control arousal and autonomic activity, also contributes to affective disturbance and exercise intolerance. Given the deep orientation of these structures in the brain, non-invasive and feasible interventions are overall lacking for neurological conditions.

As previously mentioned, SSM may improve psychophysiological processes, including motivation, the affective state, and arousal [1,2,11]. These effects may help counteract anhedonia and motivational deficits often observed in individuals undergoing physical rehabilitation. For example, Bowles et al. found that individuals undergoing exercise rehabilitation reported a higher motivation and accumulated greater walking distances while listening to music [78]. Importantly, listening to SSM during exercise rehabilitation appears to result in superior adaptations, as evidenced by improved physical activity levels, exercise capacity, and resting cardiovascular outcomes [79]. These findings parallel those shown in healthy populations. Indeed, SSM has been shown to increase motivation during a wide range of exercise modes, including rowing, weightlifting, cycling, and sprinting [7,8,9,17,23]. Thus, improvements in motivation appear to be a key translatable mechanism from healthy populations to neurological conditions, highlighting the potential use of SSM for overcoming exercise barriers.

Additionally, SSM may impart ergogenic benefits through a lower fatigue perception through affective arousal. The feedback associated with fatigue may limit exercise ability in neurological conditions, even prior to reaching true exercise capacity [80]. For example, Winward et al. showed that higher fatigue levels are associated with lower mobility and physical activity in individuals with Parkinson’s disease [81]. While the mechanisms of how music influences fatigue are still being investigated, decreases in fatigue perceptions are likely due to underlying neural changes controlling attention and affective arousal. Bigliassi et al. showed listening to music during exercise reallocates the attentional focus to external factors and increases psychological arousal with the concomitant activation of the inferior frontal gyrus [35]. Authors postulated that the activation of regions of the brain important for attention and arousal, such as the inferior frontal gyrus, may modulate interoceptive signal processing and increase arousal, which may attenuate the exercise-related conscious awareness and combat fatigue [35].This has been repeatedly confirmed in healthy and clinical populations alike, showing that affective arousal is largely influenced by SSM. For example, Rogers et al. showed that SSM results in a “psyching up” effect leading to increases in isometric strength [6]. Thus, SSM may be a useful aid in combating affective disturbances in individuals with neurological conditions through increasing motivation and psychological arousal.

### 3.4. Motor Impairments and Dyskinesia

Motor impairments and alterations in normal movement patterns are widespread across various pathological conditions, especially neurological disorders including Parkinson’s disease, Multiple Sclerosis, and cerebrovascular accidents. Frequently, motor deficits present as alterations in the gait, movement velocity, motor coordination, and postural instability [82]. This often leads to poorer health outcomes, including an increased risk for falls, cardiovascular disease, and pre-mature mortality [83]. While there are a plethora of therapeutic devices and modalities which may improve functional outcomes of motor function, many are invasive, lack formal approval for medical use, or are not translatable across populations with varying degrees of disability [84,85,86,87].

Music listening represents a cost-effective and non-invasive therapy to improve the exercise ability in individuals with motor impairments. Alterations in motor coordination and control have been widely reported with music listening across a variety of neurological conditions, including Parkinson’s disease, Multiple Sclerosis, stroke, and other neurological disabilities [88]. Indeed, neurological conditions such as Parkinson’s disease result in the deterioration in movement rhythmicity and proper gait mechanics and can result in greater trunk instability [89]. Music has been shown to improve motor control and gait patterns through alterations in neural activation and processing. Alterations in motor function are thought to be due to a series of complex interactions between motor areas of the brain, including the pre-motor cortex and cerebellum. The stimulation of these areas may aid in movement planning, motor coordination, and the special organization of ambulation [90]. However, it is likely that these effects are dependent on the selection of the type of music. For example, De Bartolo et al. showed gait pattern improvements with varying types of music genres in individuals with Parkinson’s disease [91]. In healthy populations, it has been repeatedly established that SSM results in the superior power and force development of skeletal muscle [1,5,6]. This has tremendous implications for neurological conditions with muscle weakness or at risk of falls. Indeed, individuals with a lower fall risk have been suggested to have greater lower body strength and power development [92]. SSM may allow for optimizing effort and muscular force development during rehabilitation in neurological conditions. The use of music in the context of muscular power and strength training in neurological conditions has been largely unexplored and will require further controlled studies to identify potential benefits. Although it is known that music alters the neural activation associated with movement, more research is needed to determine how SSM in particular affects mechanisms that can be leveraged to improve physical abilities in neurological conditions.

## 4. Conclusions and Future Research

The evidence continues to support music listening as an ergogenic aid in a wide variety of exercise modalities, intensities, and fitness levels. The ergogenic potential of music largely arises from psychophysiological benefits that music provides, which include but are not limited to increased motivation, autonomic responses, dissociation, mood, and muscular power. Because of the multiorgan systemic effect that music has on human health, the use of music implementation in clinical populations has increased, but the mechanistic understanding of interventions remains limited. SSM’s physiological and psychological benefits may mediate many of the mechanistic deficits that certain clinical populations may experience.

While psychophysiological benefits have been reported for many types of music, preference and self-selection appear to be key mediators of benefits and are context-dependent. For example, music that is relaxing in nature increases parasympathetic activity, while motivational music influences sympathetic activity. SSM may be used to combat symptomology which limits exercise and rehabilitation in many clinical populations. Thus, the appropriate context and personalization of music interventions likely hold the greatest potential for the translation to improved clinical outcomes in neurological conditions.

SSM has an immense potential to positively impact the limbic system, specifically that which is associated with reward and emotional regulation. By improving mood and reducing perceived exertion, not only will the psychological barriers to exercise appear diminished but the adherence to exercise programs and the enjoyment of exercise will likely increase. By shifting attention away from pain, reducing anxiety, and enhancing emotional coping strategies, neurological conditions can use SSM during exercise and rehabilitation to improve performance and reduce pain. Lastly, SSM appears to alter the neural activity in areas of the brain important for motor control and coordination. This makes using SSM appealing for use in various rehabilitative settings to potentially improve neurological conditions. A major limitation to the current review is that much of the translational knowledge included is theoretical at the present, which suggests a growing need for further investigation.

Future research is a dire need to fully realize how SSM may potentially improve neurological conditions. Arguably, the greatest need in future research is the further quantification and implementation of the mechanisms of SSM in neurological conditions. The characterization of SSM- induced mechanisms through imaging (e.g., functional magnetic resonance imaging), adaptive biomarkers (e.g., endocrine/peripheral signaling), and psychophysiological testing (e.g., autonomic arousal) in neurological conditions is warranted, and the implementation of SSM as a therapy will be difficult until more knowledge on mechanisms is apparent. It is also currently unknown as to which target populations have the greatest or most promising potential to benefit from SSM. Given that the effects of SSM influence both motor and non-motor processes, SSM is likely to have a widespread benefit, but it remains unknown if the disease status, pathological severity, medication use, or other confounders may alter the efficacy of SSM. Lastly, the use of technology to integrate SSM into practice has yet to be optimized or identified. The integration of mobile apps and wearable sensors may increase the ease of use and access while also allowing for real-time personalization and monitoring. While the current review provides a theoretical framework for how ergogenic mechanisms from SSM may translate to neurological conditions, future research and clinical trials will be necessary to confirm benefits.
